# On the Efficacy of Mercurial Action in Diseases

**Published:** 1813-12

**Authors:** D. H. Davies

**Affiliations:** 27, Carburton-street, Fitzroy-square


*To the Editor of the Medical and Physical Journal.*

SIR,

THE insertion of the following observations connected with the relation of a case in your valuable miscellany, will oblige the author.

In some former Numbers of your Journal, I have more than once attempted to point out, by the result of fortunate cases and analogous reasoning, the practicability of curing diseases, particularly those of an inflammatory kind, by inducing a new action into the system, and thus superseding the morbid one previously existing. I am well aware that the exhibition of mercury in these diseases is not a novel mode mode of treatment, nor the principle I have just noticed unknown; for the axiom on which the rationality, and, as far as my limited experience extends, the practicability of this plan of treatment rests, was laid down many years ago by the celebrated Mr. John Hunter, whose idea was that it was incompatible with the laws of the animal economy to support two actions in one system at one and the same time; but though I disclaim any merit as to the discovery of this great pathological axiom, I have endeavored, with some perseverance, for several years past, to build a practice on the knowledge of it, the striking advantages attending which have been most conspicuously apparent in a number of instances, some cases of which I have related in this Journal; and in other cases of equal success, I have solicited the attention of my professional friends to witness the success resulting from this mode of treatment. Mercury is frequently administered by medical men, yet the principle on which I conceive it to act is not generally admitted. That principle may be erroneous, but I much wish to excite professional men to a candid inquiry of the subject, as I do entertain an opinion that it has prepondency of evidence on its side. This induces me to offer some further observations on the subject, as I conceive it is upon a knowledge of this principle alone that an extended and general adoption of this practice will ensue.

Ii is my idea that the benefits resulting from the exhibition of mercury into the system in cases of deranged morbid action, particularly of an inflammatory kind, are solely attributable to the production of a new action; and that a decided and perfect remission of symptoms never takes place till the action is perfectly established; and that the system, unable to attend to the diversified operations of two actions existing at one time, the morbid one is forsaken in order to attend to the formation of secretions which the new action requires, in order to maintain its existence in the system. Under this impression of the principle on which this remedy acts, it is conclusive that the mineral ought never to be discontinued till it is evident that a new action is perfectly formed, which is evinced by a ptyalism being produced; proving that the powers of nature are all put under requisition to support and maintain for a certain period the continuance of a new and inordinate action.

In cases then of a morbid inflammatory action, such as hepatitis, enteritis, gastritis, inflammatory rheumatism, incipient stage of phthisis pulmonalis, and diseases of a similar nature, the practice of administering mercury, and stopping any thing short of this effect being produced, is *inert and*

 *useless;*

*useless;* because, not being carried to that extent as to call forth a new action, the powers of the constitution are not diverted from the maintenance of the diseased action now existing, and thus the morbid derangement continues.

What has led me to entertain the idea that the exhibition of mercury in diseases is inefficient, and, to say the least, extremely uncertain, till a new action is established, is, that I have invariably found, whilst administering this medicine, that patients have perceived no visible relief or amendment of symptoms till the ptyalism commenced; and that in the instance I shall relate, every symptom remained in its aggravated form till within a few hours of this effect being produced, and that, when it was established, the patient literally complained of little else but the unpleasant soreness of his mouth, and the inconvenience attending an increased secretion of saliva; and in other instances, as well as this, I have never found any visible remission of symptoms to any extent, till the new action was established.

I am not disposed to be a bigot to any opinion, but surely this is very conclusive of the truth of the position I have endeavored to establish, that the cure is dependant on one action superseding another. This consideration of the subject naturally leads me to another point of inquiry, viz. whether or not there is any thing specific in the nature of mercury, or that it ever acts as a specific in any one disease whatever? A great deal has been said about its assimilation with diseases, and of the kind of affinity subsisting between the disease and the mineral; but I must confess, as far as my observations have led me to judge, I feel convinced in my own mind that were I able to produce the same strength of action by any other vegetable or mineral, the same success would attend its use; and I am equally inclined to think that even in lues venerea, mercury is *no specific.* It never is of any benefit without a ptyalism being produced, and it is universally admitted that without this test no reliance is to be placed on its administration in this disease: then why may we not infer that it cures the disease by diverting the powers of the system from carrying on a continuance of the diseased action, and by establishing a fresh one? But it may be said that this reasoning is just, and the circumstances quoted may bear it out, if the first position is accurate and certain in its nature, viz. that it is inconsistent and impossible for nature properly to support two actions at one and the same time. Now, in solution of this question, analogy only can be resorted to. It first must be recollected that this is a question that has not been much canvassed or discussed, that the truth of the axiom has never been seriously inquired into, and and consequently the practice that may be reasonably built on it never fairly and impartially tried. It must particularly be remembered, in reflecting on this subject, that I do not intend to insinuate that *any* new action introduced into the system will supersede and destroy inflammatory action,—by no means, if that action is not stronger in its nature, or, more correctly speaking, capable of exciting stronger commotion or more violent excitement in the system than the disease, it falls short of the desired effect. I entertain at the same time no doubt but that a comparatively mild action, *newly* introduced into the system, may mitigate or retard diseased action, but not being able to divert the powers of nature entirely and completely from the maintenance of the morbid action, it still in some degree continues: for instance, how frequently do we see incipient symptoms, nay even aggravated symptoms of phthisis pulmonalis, suspended, as soon as the action of utero gestation has commenced in the female; but the action not being sufficiently violent to divert entirely the powers of the constitution from the disease, as soon as the suspensory powers of this action are removed by the process of utero gestation being completed, the disease resumes its accustomed powers over the system, and its ravages terminate in dissolution. Again, it is far from usual that the action of gestation ever commences while the process of suckling is going on. What can this be attributed to but the incapability of the system in supporting two distinct actions at the same time? Again, to what source are we to attribute medical men never being witnesses to two distinct unconnected diseases existing in one system at the same time, if the position I have endeavored to establish is incorrect? Why should we not hear, amongst the innumerable relation of cases on record, of instances where, for instance, scarlet fever and small-pox have existed in full force, at one and the same time, or any other two distinct diseases? I am ready to admit that, on the decline of a disease, when the stimulus of the action is comparatively worn out, a fresh disease may make its appearance; but that two diseases shall exist in full force at the same time I conceive is *impossible*, and inconsistent with the regulated laws of the animal economy; and, therefore, that in cases of morbid inflammatory action, if we are able to induce a more powerful action than the disease, (and that I think is practicable by the aid of mercury, if properly and efficaciously administered,) we shall not only suspend but destroy the disease at the time existing.

Again, analogy will not only bear this position out with respect to the accustomed and ordinary operations of nature, but also in reference to those astonishing cures that have occasionally been performed by the agency of empirical practice; and, without enlarging into a history of such cases, I shall only take notice of some cases of gout, which, if I am allowed the expression, have been dangerously cured by the administration of the Eau Medicinale, a medicine universally allowed to be composed of the most drastic and powerful ingredients. The usual result from the exhibition of this medicine in most cases has been most powerful and excessive sickness and purging. Now in my idea, though I am no advocate for such uncertain powerful medicines, yet I firmly believe that the good effects that have occasionally resulted from its use have been solely attributable to its agency in producing a new and violent action in the system, and diverting the powers of nature from the seat of the disease.

From the preceding observations, I should be sorry to be misconceived, and to be thought bigotted to this mode of practice only in the treatment of that class of diseases I have hinted at, to the exclusion of all other remedies. Such a conclusion, from what I have stated in these pages, would be totally irrelevant from my ideas on the subject.

In those cases where inflammatory action runs high, I advocate in the strongest manner the use of the lancet; and I likewise wish to mention that I conceive it a most powerful auxiliary, in the treatment of those diseases, to the mode of practice I have recommended, viz. the exhibition of mercury to the extent of ptyalism; and that I conceive the evacuation of blood disposes to a state of system peculiarly favorable to the impression of mercurial action; that even in delicate and spare constitutions it ought always to precede the exhibition of this mineral, as a much less quantity of it, under those circumstances, will affect the system so as to produce the action required. But still the great object accomplished by the exhibition of mercury in these diseases, is the possessing the power of vanquishing and subduing the disease in a shorter time, and lessening the period of human suffering; and this surely is an object worth attainment.

Having made these observations, I shall subjoin concisely the history of a case that will in some measure tend still more to elucidate the subject,

Mr. K., aged 28, of Foley-place, about two months since, applied to me for relief. When I first was requested to visit him, he had been for several days laboring under acute symptoms of pneumonia; and at this time the inflammatory state of the disease was unabated. He had applied to a medical gentleman, but had experienced no relief from what medicines had been administered. He complained of dreadful dreadful anxiety about the chest, excessive shortness of breath, the pulse was rapid and full, skin hot, tongue furred, high-colored urine, incessant cough, and little expectoration. Under these circumstances I thought it necessary to take away some blood, which was accordingly done to the extent of about twenty ounces; a blister was applied to the chest; and he took six grains of the Pil. Hydrarg. (with the addition of a little Extr. Papav. to prevent its purging) every four hours, with a dose of a saline fever mixture. Symptoms remaining in the same aggravated form the next day, I bled him again nearly to the same amount, and likewise the day succeeding, at the same time continuing the exhibition of Pil. Hydrarg. but gradually increasing the dose. This plan of treatment evidently prevented the active state of the disease increasing, but no material relief or mitigation of symptoms took place till the seventh day from the taking of the mercury. It is necessary here to observe that the bleeding evidently was instrumental in removing the active stage of the complaint, but the diagnostic symptoms of the disease still remained in full force, such as the laborious breathing and distressing harassing cough, &c. &c.; and, in addition, the incipient symptoms of phthisis pulmonalis made their appearance during the last five days—the face was flushed; the palms of the hands were affected with burning heat; the fever assumed within the last day more of the hectic form, having exacerbations in the day; the countenance assumed the hypocratic form; and the patient experienced profuse morning perspirations. On the evening of the sixth day from the commencement of the mercury, he remained in this state, with very little amendment apparently in his favor; but on seeing him next day (the seventh), the breathing was become almost natural, the cough had nearly subsided, the perspirations were not in any degree so copious, he had experienced no return of the flushes; in short, every unpleasant symptom had left him: almost the whole of his complaint *now* was the painful inconvenience resulting from the mercury having affected his mouth *pretty severely*. From this moment convalescence succeeded, and a rapid amendment was apparent; and he is now grown quite robust and hearty, and is enjoying the most perfect health.

Without again enlarging into observations, I shall leave the subject for the consideration of your intelligent readers.

D. H. DAVIES.

27, *Carburton-street,*

*Fitzroy-square.*

